# Ketogenic therapies for glioblastoma: Understanding the limitations in transitioning from mice to patients

**DOI:** 10.3389/fnut.2023.1110291

**Published:** 2023-03-07

**Authors:** Angela D. Clontz

**Affiliations:** Department of Nutrition, Meredith College, Raleigh, NC, United States

**Keywords:** glioblastoma, ketogenic diet, calorie restriction, low carbohydrate diet, diet intervention

## Abstract

Glioblastoma Multiforme is an aggressive brain cancer affecting children and adults frequently resulting in a short life expectancy. Current cancer therapies include surgery and radiation followed by chemotherapy, which due to their ineffectiveness, requires repeated exposure to the same therapies. Since the 1990s, researchers and doctors have explored other therapies, such as diet therapies, to aid in combating gliomas. The ketogenic diet has gained popularity due to Otto Warburg’s theory that tumor cells prefer “aerobic glycolysis” and cannot metabolize ketones. The inability of gliomas to use ketones provides an excellent opportunity to weaken the tumor while protecting healthy cells during cancer treatments. This review will examine some of the current research using the ketogenic diet as a form of cancer therapy to determine if this intervention is manageable and effective in patients with glioblastoma. Peer-reviewed articles from 2009 to 2019 were used. The primary objective is to distinguish differences between pre-clinical and clinical research to determine if the ketogenic diet is reproducible from mouse models into humans to determine its effectiveness. The analysis revealed several limitations of the ketogenic diet as an intervention. The effectiveness is more robust in mice than in human studies. Furthermore, tolerability is marginally supported in human studies requiring more reproducible research to validate that the intervention is manageable and effective in patients with glioblastoma.

## 1. Introduction

Glioblastoma multiforme (GBM) is a fatal form of brain cancer that affects adults and children (1, 2). It is immensely invasive due to its ability to become vascularized (2). Nonetheless, what makes GBM so deadly is its unique ability to repair damaged DNA from radiation and chemotherapy. This unique ability enables GBM to become treatment resistant creating new challenges for doctors and patients (2, 3).

Treatment for patients with GBM is a burdensome process. Standard treatment requires partial or complete tumor removal then radiation and chemotherapy for six to 9 months (1, 3). Yet, the aggressive, vascular nature of GBM leads to disease progression, otherwise known as tumor recurrence, around 6 months post-surgery and treatment (3).

When progression occurs, the protocol is to repeat the standard therapy. However, repeated exposure to radiation and certain chemotherapies, such as temozolomide, leads to inflammation and edema in the brain (2–4). These symptoms precede seizures and other neurological disorders that decrease survivability (2, 5). Therefore, researchers consider the current therapies for treating GBM antiquated and seek novel cancer treatments that prolong patient survival.

**Figure 1:**
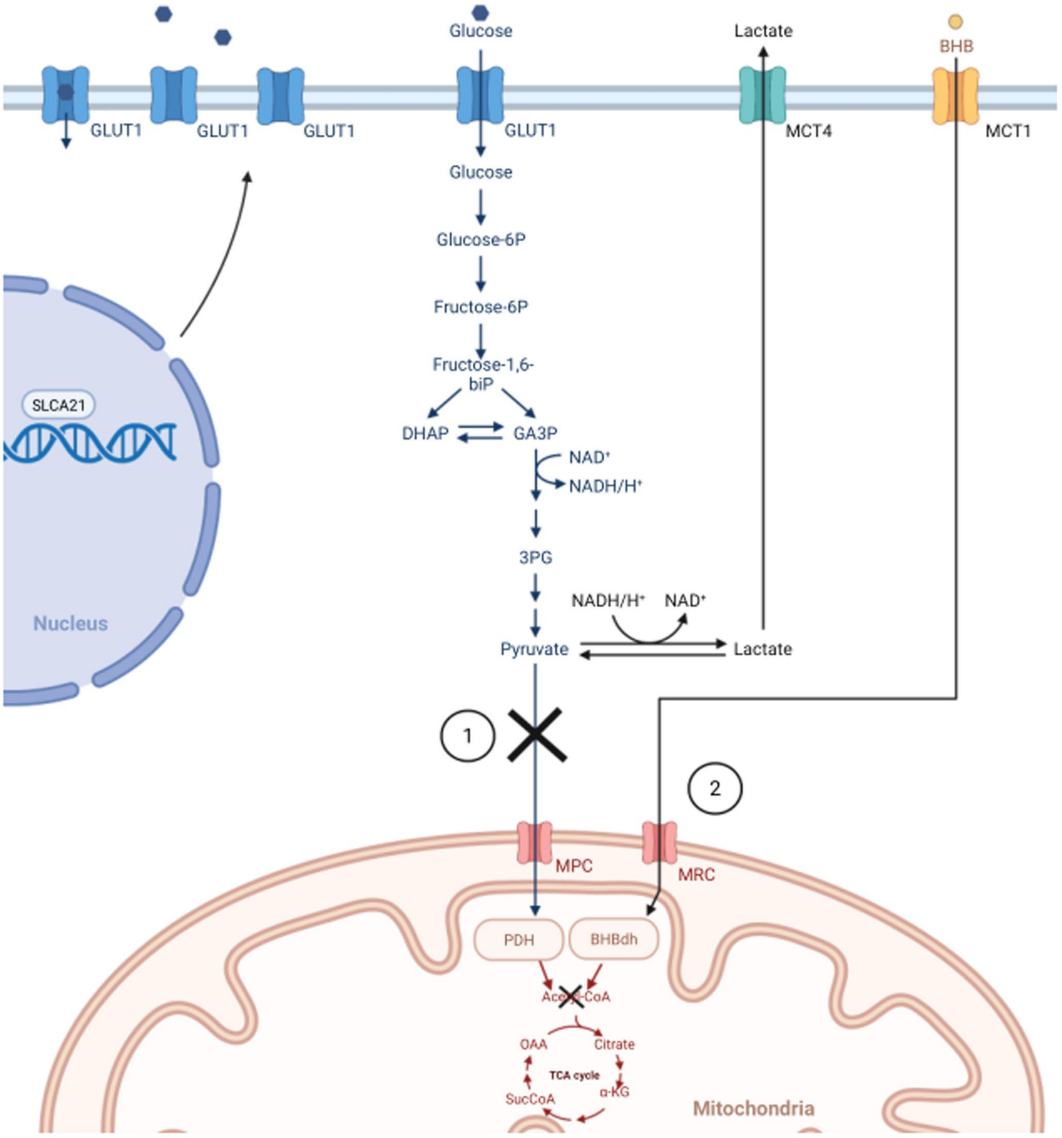
In normal cells, glycolysis only occurs in the cytoplasm under anaerobic conditions, which generates small amounts of energy. During aerobic conditions, normal cells prefer the TCA cycle and oxidative phosphorylation through the mitochondria. The Warburg Effect states that cancer cells prefer “aerobic glycolysis” for faster, safer energy production due to defective mitochondria. 1) In normal cells, pyruvate would be converted into Acetyl-CoA to start the TCA cycle. Due to dysfunctional mitochondrion (e.g., downregulated MPC, inhibition of PDH), cancer cells convert pyruvate to lactate to drive energy production. Subsequently, lactate is transported out of the cell *via* MCT, thus increasing the need for cancer cells to upregulate the SLCA21 gene to increase production of GLUT1 transporters allowing more glucose into the cell. 2) In healthy endothelial cells of the brain, BHB can be converted into Acetyl-CoA to facilitate the TCA cycle when glucose is low. Warburg states the process is not possible in cancer cells, and the reason for utilizing ketogenic diets in treating GBM. GLUT1, glucose transporter 1; DHAP, di-hydroxy acetone phosphate; GA3P, glyceraldehyde 3-phosphate; CoA, Coenzyme A; TCA, tricarboxylic acid; MPC, mitochondrial pyruvate carrier; PDH, pyruvate dehydrogenase; SLCA21, Solute Carrier Family 2 Member 1; MCT, Monocarboxylate transporter; BHB, β-hydroxybutyrate; MRC, mitochondrial respiratory complex; BHBdh, β-hydroxybutyrate dehydrogenase. Created with BioRender.com.

Scientists have been investigating cancer metabolism to understand its role in treatment resistance as early as the 1920s (6). In the 1950s, Nobel Prize winner Otto Warburg made a significant scientific discovery in cancer metabolism. Warburg discovered that cancer cells use what he termed “aerobic glycolysis” to generate energy from the conversion of glucose to lactate at high rates, even when oxygen is present (6, 7) (Figure 1). His discovery claims that cancer cells use metabolic pathways that are faster at producing energy, even though less, due to defective mitochondrial respiration—suggesting that cancer cells do not metabolize ketones (5). Warburg’s findings are well-recognized and cited throughout cancer research, and his conclusions coined the research term the “Warburg Effect” (5, 7–11). This discovery is an immense contributor to new cancer therapies, including exploring diet interventions utilizing calorie restriction (CR) and the ketogenic diet (KD) (8).

The KD is a nutritional intervention that encourages ketone production. Ketones, specifically acetoacetate and β-hydroxybutyrate, are synthesized in the liver through a process called ketogenesis. During ketogenesis, fatty acids are broken down into ketones on an as-needed basis to generate energy (12, 13). This process usually accelerates during extended periods of fasting or CR when glucose is not readily available. In addition, insulin will be reduced creating a surge of fatty acids and ultimately activating ketogenesis (14). Since the brain relies heavily on glucose for energy, it will quickly resort to ketogenesis before any other organ in the body (15). Gliomas, however, are not so quick to convert to this process as they are considered “metabolically inflexible” due to impaired mitochondrial function (5, 10).

The first reported clinical research trial testing the Warburg Effect in patients with GBM was by Nebeling et al. in 1995 (16). The study used the KD to treat two children with advanced-stage brain tumors. The results cumulated from the study showed that a KD might be a possible adjuvant diet intervention with standard cancer therapy and increase patient survival.

The outcomes of this study spurred additional diet intervention research across the globe. Most of the research on the effect of the KD on brain tumors is in pre-clinical research (mouse models). Scientists perform in-depth examinations of the high-fat, low-carbohydrate diet to understand better its effect on brain tumors (gliomas) and survival in mice. However, there are fewer human clinical studies focusing on the KD in treating gliomas, specifically GBM. It is speculated that the few number of clinical trials is due to concerns over quality of life and well-being of terminally ill patients, not just due to diet tolerability and ketosis (5).

As there is a strong need for *de novo* cancer therapies in treating patients with GBM, this review aims to explore whether the KD combined with GBM cancer therapies is manageable and effective. This review will examine pre-clinical and clinical research that used a KD in treating GBM. The outcomes being measured to determine manageability and effectiveness include diet tolerability, tumor response, disease progression, and overall survival.

## 2. Methods

### 2.1. Search strategy

A literature search was performed through the Carlyle Campbell Library at Meredith College in Raleigh, North Carolina. A list of relevant key words was curated for both ketogenic diet and glioblastoma. The search included the following key terms: “ketogenic diet” and “glioblastoma,” “ketogenic diet” and “gliomas,” “calorie restriction” and “glioblastoma,” “calorie restriction” and gliomas,” “diet intervention” and “glioblastoma,” “diet intervention” and “gliomas,” and finally, “low-carbohydrate diet” and “glioblastoma,” “low-carbohydrate diet” and “gliomas.”

### 2.2. Study selection and data extraction

All initial studies identified from the searches were saved and uploaded into Covidence (RRID:SCR_016484) for title and abstract screening. Duplicate references were removed through the screening process. All eligible studies were reviewed in full by the author. A meta-analysis performed by Klement et al. provided additional screening of selected articles to confirm eligibility (17).

Inclusion criteria for this review included only peer-reviewed, scholarly articles published in English between 2009 and 2019. Study designs were *in vivo* pre-clinical research, patient case studies, randomized controlled trials, and retrospective studies focusing on GBM being treated with a KD. Exclusion criteria included non-peer reviewed articles published before 2009, literature reviews, systematic reviews, meta-analyses, and articles that did not measure glioblastoma, ketogenic diet or low-carbohydrate diet related to tumor response or disease progression.

## 3. Results

### 3.1. Summary of search results

**Figure 2:**
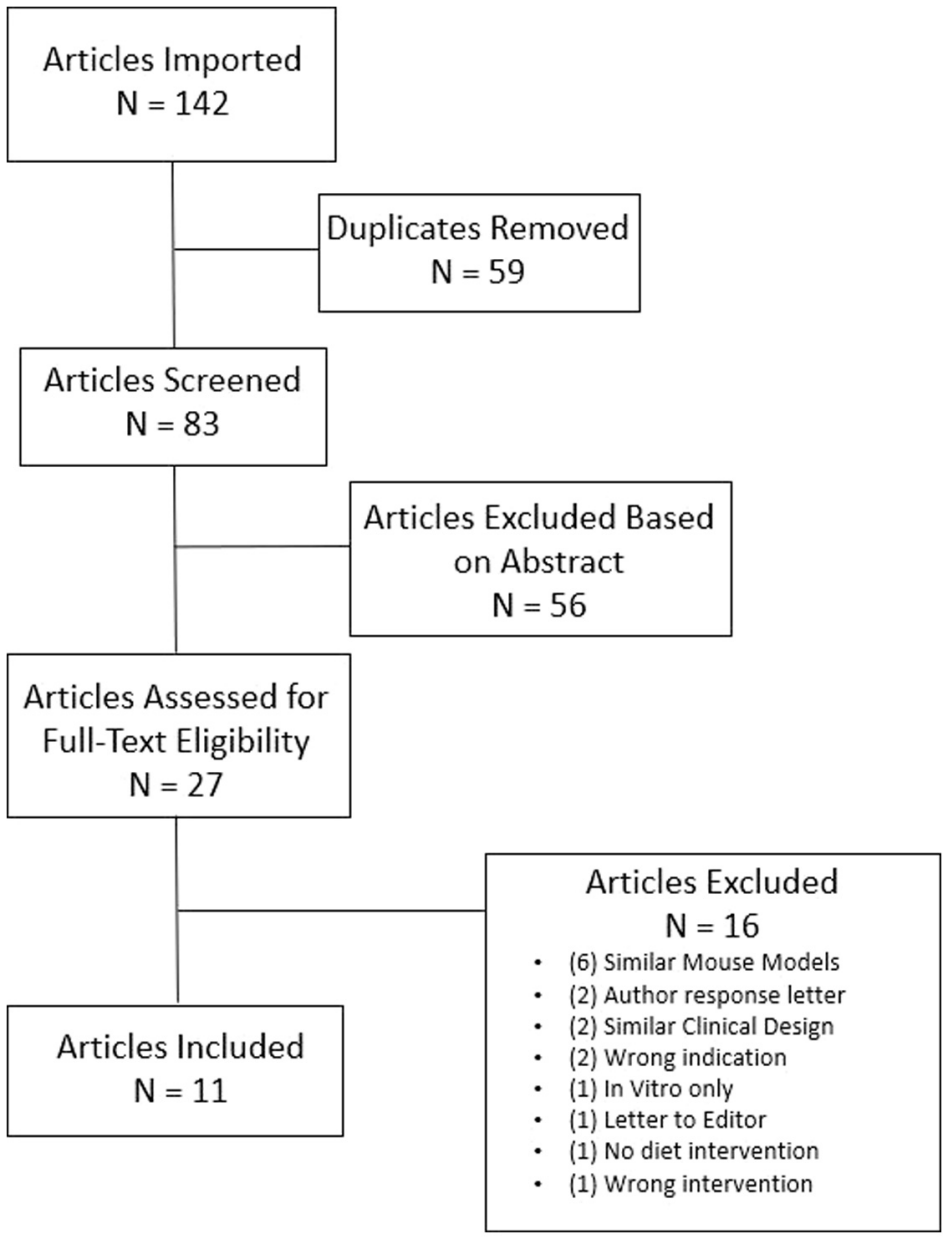
Flow diagram of search process. *N* is an abbreviation for number. Searches include: “ketogenic diet AND glioblastoma,” “ketogenic diet AND gliomas,” “calorie restriction AND glioblastoma,” “calorie restriction AND gliomas,” “diet intervention AND glioblastoma,” “diet intervention AND gliomas,” “low-carbohydrate diet AND glioblastoma,” “low-carbohydrate diet AND gliomas.” All initial studies retrieved from the search were uploaded to Covidence for title and abstract screening and reviewed for eligibility per outcomes being measured for the review.

A total of 142 articles were identified with 59 duplicates removed. Eight-three abstracts were screened for relevance to measures and outcomes of interest with 56 studies deemed irrelevant. Twenty-seven articles were full text reviewed to confirm eligibility. After further review, 16 articles were considered not eligible thus leaving 11 articles that qualified for this review (Figure 2). Of the 11 articles included for this review, four were animal studies and six were human studies with one study combining both animal and human trial data. The total number of GBM patients treated with a KD, or low-carbohydrate diet, was 76 and the total number of mice was 190 (Table 1).

**Table 1:** Study characteristics of articles investigating the KD in treating GBM.

Author	Country	Study purpose	Study type	Study population	Cell line	Study design	Max duration	Macronutrient composition	Conclusion
Abdelwahab et al. (7)	United States	To investigate the efficacy of a KD using KetoCal^®^ and radiation therapy to treat malignant gliomas.	Mouse	*n* = 60	GL261	Mice implanted with gliomas then randomized to SD or KetoCal^®^ groups with or without radiation.	299 days	72% fat 15% protein 3% carbs	Mice fed KetoCal^®^ alone had increased survival compared to those fed SD. KetoCal^®^ plus radiation demonstrated absence of tumor growth in 80% of mice.
De Feyter et al. (18)	United States	Brain tumor tissue would show reduced oxidative ketone metabolism compared to non-tumorous brain tissue; brain tumor growth would be slower when mice are fed a KD and survival would be longer.	Mouse	*n* = 40	9 L and RG2	Mice implanted with gliomas and randomized to KD or SD; MRI and MRS were used to assess tumor growth.	50 days	91% fat 9% protein 0% carbs	Gliomas can metabolize ketone bodies. No difference observed in ketone oxidation between gliomas and normal brain cells. KD did not demonstrate a positive effect in survival.
Lussier et al. (20)	United States	To investigate an unrestricted KD in alleviating tumor immune suppression in a malignant glioma.	Mouse	*n* = 34	GL261	Mice implanted with gliomas and depleted of CD8 T cells; Randomized to 4:1 KetoCal^®^ or SD; bioluminescence to measure tumor burden.	60 days	72% fat 15% protein 3% carbs	A KD may be feasible by reducing immune suppression and promoting immune mediated killing of the glioma. The use of a KD as adjuvant therapy with current and newer therapies for treating gliomas is supported by this data.
Mukherjee et al. (19)	United States	To determine if a KD paired with a glutamine antagonist could suppress tumor growth and prolong survival.	Mouse	*n* = 24	VM-M3 and CT-2A	Mice received KetoGEN with calorie restriction and glutamine antagonist.	15 days	89.2% fat 8.7% protein 2.1% carbs	The benefits of using a glutamine antagonist in combination with a restricted KD can stop glioma cell growth and promote survival in mice.
Rieger et al. (24)	Germany	To investigate if KD affected efficacy of bevacizumab.	Mouse	*n* = 32	U87MG	Mice randomized to KetoCal^®^ or SD and given chemotherapy twice a week.	28 days	72% fat 15% protein 3% carbs	KD showed strong effect to chemotherapy and affecting ATP levels in the tumor.
Elsakka et al. (10)	Egypt	To demonstrate a KD with hyperbaric oxygen therapy and calorie-restriction enhance metabolic efficiency in normal brain cells while inhibiting tumor metabolism.	Human	*n* = 1 38-year old male	GBM	Individual case study of calorie-restricted KD with hyperbaric oxygen therapy, radiation, and chemotherapy. MRI and MRS to measure tumor growth and assess metabolites.	20 months	71% fat 22% protein 7% carbs 900 kcals/day	KD reduced lactic acid fermentation that inhibited tumor growth as well as reduced inflammation and edema in the brain. This environment reduced tumor invasion after surgery. Increased ketone bodies fueled and protected the patient’s normal cells from the oxidative stress of radiation/chemotherapy treatment.
Woodhouse et al. (5)	United States	To assess the feasibility and safety of the MAD in attaining ketosis in patients with gliomas.	Human	*n* = 29 males = 17 females = 12 ages = 30–76	GBM grade II-IV	Retrospective study that assessed medical records for patients with documented ketone values who received the MAD.	24 months	<20 g carbs/day	MAD is feasible and safe during radiation and chemotherapy treatment for patients with gliomas. Ketones may be a radiation sensitizer especially in non-methylated GBMs.
Zuccoli et al. (1)	Italy	To determine if a restricted KD combined with standard cancer therapy is effective in managing tumor growth.	Human	*n* = 1 65-year-old female	GBM grade IV with MGMT gene	Individual case study of a patient who completed a calorie-restricted KD (KetoCal^®^) and chemoradiation therapy.	13 months	72% fat 15% protein 3% carbs 600 kcals/day	GBM treated with a restricted KD showed evidence of rapid regression of disease following surgery.
Artzi et al. (11)	Israel	To detect and characterize changes in brain metabolites in patients with high grade gliomas treated with a KD using ^1^H-MRS.	Human	*n* = 9 male = 5 female = 4 ages = 27–69	Gliomatosis Cerebri and GBM	Patients with GBM treated with KetoCal^®^ and chemotherapies, then scanned with ^1^H-MRS every other month to detect ketone metabolism until off study.	31 months	72% fat 15% protein 3% carbs	Two out of 5 patients with KetoCal^®^ had brain tissue ketone detection. Patient with gliomatosis cerebri had a clear shift from glucose to ketone metabolism with KD alone. Inconclusive findings in other three patients. One patient had ketone detection within the tumor. Diet compliance was intermittent.
Champ et al. (8)	United States	To assess the safety of a KD, and its effect on glucose levels, when used as adjuvant therapy with radiation and chemotherapy.	Human	*n* = 6 ages 34–62	GBM grade III-IV	Retrospective medical record review of patients with high grade gliomas with documented glucose levels and serum ketone levels who consulted the treating physician about the KD as a form of treatment.	12 months	77% fat 15% protein 8% carbs	KD is considered safe and well-tolerated as an adjuvant therapy to cancer therapies. Restriction of carbohydrates to reduce glucose levels may improve treatment response and overall survival. The average time on diet was 7 months. 2 out of 6 patients had tumor recurrence.
van der Louw et al. (23)	Netherlands	Assess the feasibility and safety of the KD as a treatment option for patients with recurrent GBM.	Human	*n* = 10 male = 9 females = 1 ages = 33–65	GBM	Open-label, non-randomized study of recurrent GBM after surgical resection and chemoradiation. Patients received liquid and solid KDs.	14 weeks	Liquid: 88.8% fat 8.2% protein 2% carbs Solid: 88.4% fat 7.6% protein 8% carbs	60% diet compliance. Ketosis was reached within a week with no major side effects. The use of a KD as adjuvant therapy to standard cancer treatment, is feasible and safe in patients with GBM.
Rieger et al. (24)	Germany	To investigate the safety and tolerability of a KD in patients with recurrent GBM.	Human	Human: *n* = 20 male = 7 females = 13 ages = 30–72	Human = GBM	Open-label, pilot study with recurrent GBM greater than 6 months post-surgery and greater than 3 months post-radiotherapy.	16 weeks	Human: <60 g carb/day	KD is safe and tolerable in treating patients with GBM. Ketosis is not achieved indicating a genetic or unknown factor affecting the patients from achieving this state.

*n*, number; KD, ketogenic diet; SD, standard diet; ^1^H-MRS, proton magnetic resonance spectroscopy; MAD, modified Atkin’s diet; GBM, glioblastoma multiforme; MRS, magnetic resonance spectroscopy.

### 3.2. Analysis of the KD to treat mice with GBM

Since using the KD in treating patients with GBM is still exploratory, most diet intervention research is conducted in mouse models to understand how the diets affect gliomas. Specifically, to the KD, many variations have been tested in mice to determine efficacy. Of the five mouse studies examined, variations included a liquid KD, calorie-restricted KD (CR-KD), and an unrestricted KD. Even with the studies utilizing different percentages of fat, protein, and carbohydrates, the ratio stayed around 4:1 (4 grams of fat to 1 gram of protein and 1 gram of carbohydrates). The macronutrients averaged 80% fat, 15% protein, and 5% carbohydrates across the animal studies (7, 18–20).

Tumor response while treated with a KD is a significant outcome to measure in mice. Most of the animals treated with a KD showed noteworthy results regarding tumor response. For instance, Abdelwahab et al. and Lussier et al. reported increased tumor response in mice treated with the KD compared to a standard diet (SD) (7, 20). Abdelwahab et al. included radiation treatment with either the KD or SD, which simulates typical treatment in humans. Both researchers also used a liquid formula called KetoCal^®^ that is generally provided to patients with epilepsy to help minimize seizures.

Nine out of 11 mice that Abdelwahab et al. treated with radiation and KetoCal^®^ showed satisfactory tumor response with no evidence of tumor recurrence for 104 days. Tumor burden was measured using bioluminescence with injections of luciferase prior to imaging. One mouse in the KetoCal^®^ without radiation group experienced no tumor burden for 200 days before sacrifice. The tumor response for all mice on the KD, with or without radiation, was more compelling than the SD groups with the same treatment. These results display a possible correlation that the KD could enhance tumor response for patients when receiving radiation treatment.

Lussier et al. investigated whether an unrestricted KD could enhance immunotherapy treatment in mice implanted with GL261-Luc2 tumor cells (20). The mice were depleted of CD8 + T lymphocytes before tumor implantation and diet intervention to decrease immune function. Researchers designed four treatment groups that received either a KD or an SD with or without CD8 + T cells. Both groups on the KD with or without CD8 + T cells had decreased tumor growth reported by bioluminescence compared to both SD groups. Of note, the mice in the KD group with CD8 + T cells exhibited increased CD8+ T cell production that infiltrated the tumor. These results support the notion that a KD can enhance immune function and tumor recognition when treated with immunotherapies.

Since several mouse models support the effectiveness of a KD as a treatment for gliomas, the replication of these outcomes continued to reinforce the theory of the Warburg Effect (5, 7–11). However, in a study by De Feyter et al. ketones were found in glioma cells wavering that gliomas can metabolize ketones (18).

De Feyter et al. tested whether gliomas could utilize ketone bodies for energy production *in vitro* and *in vivo*. In specific, 9 L and RG2 tumor cells were tested. Mice received either a KD with CR or an SD. The researchers incorporated CR to induce high levels of ketones in the brain.

*In vitro*, De Feyter et al. exposed RG2 tumors to high levels of ketones and low levels of glucose. Analysis showed the cells demonstrated β-hydroxybutyrate oxidation but at a low rate. This finding indicated that at least the RG2 cell line may be able to metabolize ketones. When the same *in vitro* technique was applied to the 9 L cell line, the tumor cells could not metabolize ketones.

On the contrary, the animal studies that De Feyter et al. performed showed different results from the *in vitro* experiments. Both 9 L and RG2 cell lines implanted into the animals in the KD with CR group showed a high presence of ketones when compared to the SD group. De Feyter et al. investigated MCT1 since this is a transporter that allows the passage of lactate, pyruvate, and ketones into a cell. The researchers acknowledged a “stronger MCT1 immunoreactivity was observed in the RG2 gliomas when animals were fed the KD” (18).

Due to the discrepancies between the animal studies and cell cultures, the *in vitro* testing was repeated, exposing cells to low glucose with ketones or just low glucose. The second analysis showed that neither group demonstrated defined β-hydroxybutyrate oxidation.

The animals were examined for the effectiveness of the intervention on tumor growth and prolonged survival. De Feyter et al. reported the diet intervention did not affect tumor growth or overall survival based on imaging results and immunostaining for Ki-67 (a tumor proliferation biomarker). This discovery may be a case to reject the Warburg Effect theory indicating that glioma cells could use glycolysis or β-hydroxybutyrate oxidation for energy production and proliferation.

Further exploration into other forms of cancer metabolism was examined by Mukherjee et al (19). Their testing expanded beyond glycolysis and β-hydroxybutyrate oxidation in gliomas. Several references noted by Mukherjee et al. mention that gliomas utilize glucose and glutamine for energy production. They further conclude that cancer therapies increase glutamine in tumor cells allowing the cells to utilize the glutamine-glutamate cell cycle to generate energy readily.

Mukherjee et al. experimented with VM-M3 and CT-2A cell lines, which differed from the cell lines De Feyter et al. examined. According to Mukherjee et al., these cell lines have higher incidences of spontaneous and rapid growth throughout the brain, and the reason for use in the experiment. The researchers also used 6-diazo-5-oxo-L-norleucine (DON), which inhibits several enzymes and metabolic pathways, including the enzyme required for glutaminolysis, in gliomas. Concurrently, the mice received a CR-KD to reduce glucose further (19).

After three consecutive experiments using an SD with or without DON or a CR-KD with or without DON on VM-M3 cells, the mice Mukherjee et al. treated with the intervention and DON had the highest tumor response and overall survival compared to both control groups (44% compared to 19%). These findings indicate that the KD assisted in decreasing glucose in combination with the glutamine antagonist to stop tumor growth. In the CT-2A cells, the groups that received the intervention with DON saw reductions in cell growth and increased cell arrest. These results support the Warburg Effect theory and suggest that glutamine is vital in glioma survival. In the case of De Feyter et al., this research could speculate that specific cell lines of gliomas are potentially susceptible to β-hydroxybutyrate oxidation or are better equipped to adapt to their changing environment to survive.

Even though tumor response is a primary outcome of many mouse models to determine the effectiveness of a treatment or intervention, prolonged survival is just as crucial as a secondary objective. Once implanted with glioma cells, mice typically only survive 15 to 18 days without treatment or intervention (7). For a mouse to survive beyond this time is considered a significant finding. The mouse research discussed in this review demonstrated that after implantation with gliomas and exposure to a KD, with or without other interventions, survival beyond 18 days was observed to support prolonged survival with a KD as therapy.

Many mouse models provide enough support for the Warburg Effect theory and that the KD is an effective treatment for gliomas. The KD combined with radiation therapy may be an ideal adjuvant treatment for patients with GBM. With these favorable mouse results, research branched out into the clinical setting. Can these same favorable results be replicated in clinical trials and demonstrate that a KD is a beneficial and effective therapy for patients?

### 3.3. Analysis of the KD to treat patients with GBM

In the seven human trials examined for this review, tumor response, disease progression, and overall survival were the primary outcomes to determine efficacy. These outcomes were similar in the mouse models, but, the results varied and were less prominent in the human trials. One possible reason is that mice have no choice in which diet or treatment they are given, and their environment is easily controlled and manipulated for conformity to the intervention. Human trials are more complex than mouse studies because multiple variables exist, and human genetics may be an outlier in treatment intervention. But simply, safety and tolerability are the main factors when experimenting with diet therapy in terminally ill patients.

The first study using a KD as an intervention in an adult patient with GBM was presented by Zuccoli et al (1). The patient in the single-case study was administered a CR-KD and reported no issues or complaints. However, laboratory values indicated a safety concern. In this intervention, a patient underwent treatment for GBM that included surgery, radiation, and the drug temozolomide (TMZ). The patient was given a KD that consisted of 600 kcals/day before starting radiation therapy and chemotherapy.

After 35 days, the diet changed to CR only due to high uric acid levels posing a safety concern. Even with stopping the KD and only continuing the CR, the patient did not exhibit tumor recurrence until 3 months after stopping the CR (1). This study supports one patient experienced diet tolerability, but ketoacidosis is still a safety concern. Yet, many of the articles included in this review support high levels of ketones for optimal results with standard cancer therapies.

As clinical trials start to show favorable tolerability results, a small retrospective study conducted by Champ et al. evaluated the safety of the KD and its effect on glucose levels (8). The study looked at patients with grade III-IV GBM who were given a KD with chemoradiation between March 2010 to April 2013. The patients completed the KD before, during, and after chemoradiation treatment. In addition to MRI data to verify tumor response and disease progression, they used the Revised Assessment in Neuro-Oncology (RANO) scale to provide additional data on tumor response and overall survival. The RANO scale was developed around 2011 and uses qualitative and quantitative analysis of MRI results to measure tumor size in response to treatments (22).

Champ et al. indicated that six patients out of 53 qualified for the analysis as determined by verifiable blood glucose values and being on a KD. The average blood glucose value prior to starting the KD was 127.7 mg/dl and dropped to 92.3 mg/dl during treatment. Moreover, three out of six patients received steroids during the treatment that may have adversely affected glucose levels. The control included patients on an SD who received chemoradiation therapy during the same period. The SD group had an average blood glucose value of 122 mg/dl during radiation therapy.

Even with only minor decreases in glucose levels, Champ et al. reported no adverse events or diet intolerability. However, only four out of six patients had confirmed ketosis by urine tests. For ketosis to be verifiable, blood glucose values must be <100 mg/dl and urine ketones ≥2 mmol/L (11, 17). Furthermore, Champ et al. mentioned that one patient included in the analysis opted to do CR with a KD. This patient had confirmed disease progression after 1 month of the diet therapy, but the disease later resolved without further requirement of chemoradiation through 12 months post-intervention.

Champ et al. further concluded that the average survival rate of the patients observed was 14 months post-treatment and intervention, and the average time to progression was around 10 months. These results support the KD affecting glucose levels in gliomas, even without the patient reaching a state of ketosis, for improved tumor response to cancer treatments.

Another notable clinical trial by van der Louw et al. involved a cross-over study design examining patients with GBM on two types of KD (23). The diet intervention began with liquid KetoCal^®^ (4:1 ratio) at 2,400 kcals/day for 8 weeks, followed by a solid KD (2:1 ratio, which is 2 grams of fat to 1 gram of protein and 1 gram of carbohydrates) at 2,835 kcal/day for an additional 6 weeks. The study focused on safety and tolerability of a liquid KD versus a solid KD with chemoradiation. Van der Louw et al. determined tolerability would be achieved if patients had 60% diet compliance over the 14 week-intervention by reviewing food diaries. Safety was monitored by levels of glucose and urine ketones. Coping questionnaires were provided to the patients to measure their quality of life while on the diet.

Van der Louw et al. enrolled 11 patients but only nine achieved full ketosis as determined by urine testing. Six of the nine patients maintained diet compliance while completing the study, and four continued the KD while receiving a second chemoradiation treatment. No adverse events were reported during the study. However, responses to the coping questionnaire indicated that patients, and their partners, found the solid KD intervention challenging to maintain. Patients also reported nutrition counseling during the study was needed for motivation to continue the diet therapy rather than for support to maintain defined calories and macronutrient percentages.

Even with low coping scores, van der Louw et al. reported that the average survival of patients on the diet intervention was 12.8 months. This data supports that a KD is safe and mildly tolerable but does improve life expectancy this data supports that a KD is safe, mildly tolerable, and that diet therapy may improve life expectancy (2, 5). If patients with GBM can overcome the difficulties of maintaining a KD, this may allow enough time for ketones to develop to suppress tumor growth and protect brain tissue from chemoradiation (5, 10). Better response rates to cancer treatments decrease disease progression and increase survival.

In 2007, a pilot study was conducted by Rieger et al. that focused on feasibility of a KD therapy for patients with GBM (24). The study concluded in 2011; therefore, is included in this review. Rieger et al. evaluated the safety and tolerability of using a KD without CR in GBM patients who had disease progression. This study was intriguing as it included multiple aims and multiple qualitative and quantitative measurements in the study design.

The patients included in the study had to have shown disease progression within 6 months of surgery and within 3 months of receiving radiation or relapse during or after chemotherapy. Patients were placed on a low-carbohydrate diet with meal planning and recipes to guide them. The patients were allowed to eat to satiety but could only consume at least 60 g/day of carbohydrates. In addition, patients had options to consume yogurt drinks with a 3:2:1 ratio of grams of fat to protein to carbohydrates as needed.

Of the 20 patients enrolled, Rieger et al. reported that three patients discontinued early due to diet intolerability. Eight patients stayed on the diet intervention while undergoing additional cancer therapy. The remaining patients stayed on the diet intervention through the efficacy period but later stopped the diet. At least 92% of the patients had confirmed urine ketosis at least once during the intervention. Moreover, two patients had stable disease (no progression) after 6 weeks on the diet, while one presented with a minor tumor response considered related to the diet.

Rieger et al. also evaluated overall survival; the average survival after therapy was 32 weeks. These results are slightly less encouraging than van der Louw et al., who reported an overall survival of about 12.8 months, and Champ et al., who reported survival through 14 months. As Rieger et al. indicated, some of the patients discontinued the intervention thus possibly contributing to the lower average in survival. This information may indicate that a low-carbohydrate diet induces ketosis for better tumor response to treatments.

Another cross-over study by Elsakka et al. investigated a CR-KD followed by an unrestricted KD in treating a patient with low-grade GBM (10). This study added hyperbaric oxygen therapy (HBOT) that generates oxidative stress on the glioma. The CR-KD was a 4:1 ratio with 900 kcal/day; at 9 months, the diet switched to a KD without CR of 1,500 kcal/day. In addition, the patient received a total of 20 sessions of HBOT post-surgery.

Elsakka et al. reported that the 38-year-old patient had a partial resection of a low-grade glioma after a 21-day CR-KD. The patient was further treated with chemoradiation followed by TMZ for 6 months. Afterward, the patient switched to the unrestricted KD for 10 months. The patient’s ketone levels averaged 2 mmol/L, and glucose levels averaged 65 mg/dl. At 24 months, the MRI results showed that the residual tumor decreased in size by 1.5 cm, and the patient was still alive with no disease progression. These results suggest that a cross-over CR to unrestricted KD in combination with HBOT may enhance tumor response and increase survival. The downside is that low-grade gliomas are noted to have better treatment responses than higher-grade gliomas (11).

In 2019, a different diet intervention emerged in treating patients with GBM. As there were still concerns about patient tolerability and safety, Woodhouse et al. experimented with a modified Atkin’s diet (MAD) (5). The study assessed if MAD could aid cancer therapy to reduce repeated exposure to chemoradiation. This cancer treatment is known for causing pseudoprogression. Pseudoprogression is swelling and inflammation in the brain and around the tumor due to repeated doses of chemoradiation that can lead to decreased survival (3).

Woodhouse et al. enrolled 29 patients between the ages of 30 and 77. The MAD was a 1:2 ratio of fat to protein with <20 g/day of carbohydrates. The purpose of the 1:2 ratio was to test a more manageable diet that patients could tolerate while allowing them to achieve ketosis to reduce inflammation.

Woodhouse et al. defined ketosis as the presence of ketones > 1 mmol/L, which was lower than the requirements for the study conducted by van der Louw et al. Even with 79% of the patients achieving ketosis by this value, 17% had disease progression. However, overall survival showed that 26.7% of the patients were still alive 2 years post-treatment. This survival time was the longest reported in all clinical trials included in this review. These results support that dietary interventions with calorie or carbohydrate restriction can be tolerated and could increase overall survival in terminally ill patients.

## 4. Discussion

### 4.1. Limitations in determining the effectiveness of a KD in treating patients with GBM

The pre-clinical and clinical research articles compiled for this review demonstrate support for the KD in treating patients with GBM. Mice demonstrated positive tumor responses to the KD, with some mice experiencing prolonged survival. The handful of clinical trials or human research studies showed positive results toward tolerability, tumor response, and prolonged survival. However, the methods used in these studies were equivocal as definitive support for a KD as adjuvant therapy to cancer treatment. In the pre-clinical and clinical studies, the glioma cell lines, diet interventions, glucose levels, and ketone levels were not all consistent to draw a valid correlation (Table 2). Other factors, such as genetics and glioma grade, may affect treatment efficacy with diet and drug interventions.

**Table 2:** Summary of Limitations and Inconsistencies Between Pre-Clinical and Clinical Research Treating GBM.

Author	Country	Study type	Diet intervention	Limitations	Inconsistencies observed between studies	Advantages
Abdelwahab et al. (7)	United States	Mouse	KetoCal^®^	High glucose levels Low to moderate ketone levels Non-aggressive cell line Intervention started 4 days after implantation	Different glioma cell line	Used radiation representative of standard cancer treatment seen in clinical setting
De Feyter et al. (18)	United States	Mouse	91% fat diet with 0% carbs	High glucose levels Non-aggressive cell line	Different glioma cell line Different macronutrient diet Gliomas were able to utilize ketones No effect on survival	Intervention started 7 days post-implantation that would better represent late-stage tumor growth as seen in humans High ketone levels
Lussier et al. (20)	United States	Mouse	KetoCal^®^	Intervention started 4 days after implantation Glucose and ketone levels not reported	Focused on immunotherapy Used immunodeficient mice Measured tumor response but not overall survival	None
Rieger et al. (24)	Germany	Mouse	KetoCal^®^	Glucose levels not reported Low ketone levels	Different glioma cell line	Intervention started 7 days post-implantation that would better represent late-stage tumor growth as seen in humans Used chemotherapy representative of standard cancer treatment seen in clinical setting
Mukherjee et al. (19)	United States	Mouse	KetoGEN	Intervention started 4 days after implantation High glucose levels Low ketone levels	Used DON Different glioma cell line Used calorie restriction with KD Different macronutrient diet	Glutamine antagonist helped stop tumor growth in combination with KD
Elsakka et al. (10)	Egypt	Human	Comparable to KetoCal^®^ used in mice	Single-case study Added another therapy that may not be available in all clinical settings	Used calorie restriction and water fast with KD Used HBOT	Intervention reduced inflammation and edema in the brain Low glucose levels High ketone levels
Woodhouse et al. (5)	United States	Human	MAD	Glucose levels not reported Retrospective study Some patients had MGMT gene	Different macronutrient diet Intervention only used during chemotherapy Some patients had low-grade GBM	Moderate to high ketone levels Reported diet as tolerable
Zuccoli et al. (1)	Italy	Human	KetoCal^®^	Single-case study Patient had MGMT gene Patients received steroid treatment	Used calorie restriction and water fast with KD Switch patient to calorie restriction only	Low glucose levels Moderate to high ketone levels Reported tumor response and survival
Artzi et al. (11)	Israel	Human	KetoCal^®^	Small sample size Patient with low-grade glioma demonstrated favorable response to treatment Glucose levels not reported Not all patients were consistent with intervention	Patients started intervention at different times in relation to chemotherapy Treated a patient without GBM and included in results	High ketone levels
Champ et al. (8)	United States	Human	Comparable to KetoCal^®^ used in mice	Small sample size Retrospective study Moderate to high glucose levels Low ketone levels Patients received steroid treatment	Not all patients had reported ketone levels Patients started intervention at different times in relation to chemotherapy	Diet reported as tolerable Reported overall survival and tumor response with RANO
Rieger et al. (24)	Germany	Human	< 60 g carbs/day	Pilot study Patients received steroid treatment Low ketone levels Moderate to high glucose levels	Different macronutrient diet Reported genetics or unknown factors affected inability to achieve ketosis	Diet reported as tolerable
van der Louw et al. (23)	Netherlands	Human	KetoCal^®^ and 2:1 ratio KD	Small sample size Non-randomized 60% diet compliance Moderate to high glucose levels	Used two different types of KD Different macronutrient diets	High ketone levels

KD, ketogenic diet; GBM, glioblastoma multiforme; HBOT, hyperbaric oxygen therapy; DON, 6-diazo-5-oxo-L-norleucine; MAD, modified Atkins diet; RANO, Revised Assessment in Neuro-Oncology; MGMT, O6-methylguanine-DNA methyltransferase.

Several cell lines of gliomas can be tested in cell culture and mouse models. However, animal research is expensive and cumbersome. Specific cell lines of gliomas are more expensive than others, especially the aggressive cell lines common in humans (25). Scientists may need more funding to acquire these aggressive cells for adequate research. For example, most of the cell lines used in the mouse studies were GL261, 9 L, and RG2. The most aggressive human glioma cell lines, not used in the mice, are LN229, SNB19, U87, and U251.

According to Burden-Gulley et al., the best comparable glioma cell lines are LN229 and CNS-1 or any other cell lines obtained from human cancer tissue (26). Without testing these more human-relatable cancer cells, the effectiveness of a KD in patients with GBM is not adequately represented for reproducibility. Researchers should consider using genetically engineered mice that may enhance results that are reproducible in humans (27).

Shelton et al. conducted research in mice using highly invasive VM-M3 and CNS-1 glioma cells that could represent more reproducible results for patients with GBM (28). The results of their research provided support for the genetic and metabolic characteristics of human gliomas.

Of the mouse models included in this review, Mukherjee et al. tested the VM-M3 and CT-2A cells in mice. These gliomas were targeted as comparable cell lines for reproducing diet interventions and cancer therapy in patients with GBM. In addition, Mukherjee et al. also started the KD seven days after implantation of the gliomas to provide a more realistic biomarker for human disease onset. Some of the mouse models started the KD before tumor implantation and others immediately after recovery from the implantation (17). The presence of high ketones and low glucose to a newly implanted tumor may have a significant effect on tumor growth in an *in vivo* environment. Early glioma development in a human with high ketones and low glucose is most likely a rare occurrence.

Just as in mice, there are many factors that can affect the effectiveness of a KD in treating patients with GBM. Of the articles reviewed, a handful indicated that some patients with GBM had a genetically modified strain. This variation is the presence of a promoter gene called O6-methylguanine-DNA methyltransferase (MGMT) that turns off transcription for DNA repair in tumors when exposed to radiation or chemotherapy (5, 29). If MGMT genes are present in patients, the glioma will respond to chemoradiation with or without a diet intervention (30). This modified version of GBM can elicit a false positive result in diet intervention therapies with cancer treatment.

Another inconsistency observed in the pre-clinical research was the lack of standard cancer therapy. All but two of the mouse models did not use any standard cancer treatment (radiation or chemotherapy) to treat the mice. The KD was the only intervention used to treat the gliomas. In patients, this will not be a likely scenario. Patients diagnosed with GBM will most likely undergo treatment with surgery, radiation, and chemotherapy (1, 3, 5, 8, 10, 23). As the mouse models mostly used non-human cell lines, and researchers can control an animal’s diet and environment, this monotherapy intervention may be suitable for mice but is not an accurate representation of real-life events.

Only one study was found for this review that evaluated a patient who was solely treated with a KD. Artzi et al. assessed ketone metabolites in the brain of patients with gliomas when treated with a KD (11). Out of the nine patients in the experiment, only one did the KD alone without standard cancer therapy. This intervention may have been chosen or recommended because the patient had a low-grade glioma that cannot be treated with the same GBM cancer therapy. This patient did maintain stable disease throughout the diet intervention. However, the KD alone did not decrease the tumor size or prolong survival.

The primary outcome assessed by Artzi et al. was to determine if gliomas can metabolize ketones. As most clinical KD studies monitored ketone levels *via* urine tests, this study experimented whether urine ketones could accurately represent total cerebral ketones. The study used proton magnetic resonance spectroscopy (^1^H-MRS) to take images of the brain to determine if ketones exist either in the brain or in the tumor. Artzi et al. showed three patients maintained high levels of ketones (at least 2 mmol/L) according to urine tests, but ^1^H-MRS detected no ketones in the brain.

Mouse models show favorable results with average serum ketone levels of 1.9 mmol/L. The urine ketone levels in patients averaged 1.8 mmol/L. The inconsistent ketone testing between both types of research leads to a mixed review on this topic. Based on the compiled information between the pre-clinical and clinical interventions, the KD may be more effective in treating GBM if patients can safely achieve serum ketone levels of 3 mmol/L or higher.

Ketone levels were one of the inconsistent findings making the effectiveness of this intervention challenging to assess. Glucose values were also not consistent (Table 3). As the low-carbohydrate and high-fat diets were aimed to deplete circulating glucose levels available to gliomas, not all mouse models included these values. For the studies that did, the glucose levels were between 90 and 160 mg/dl. It was not discussed if the levels in mice were comparable to human levels.

**Table 3:** Glucose, ketone, and insulin levels while using the KD in treating GBM.

Author	Country	Study type	Glucose levels	Ketone levels (BHB)	Insulin levels	KD methods
Abdelwahab et al. (7)	United States	Mouse	Post-implantation Day 6 KD = 160 mg/dL KD + Radiation = 140 mg/dL Day 13 KD = 150 mg/dL KD + Radiation = 160 mg/dL	Post-implantation Day 6 KD = 1.3 mmol/L KD + Radiation = 2.2 mmol/L Day 13 KD = 1.2 mmol/L KD + Radiation = 1.6 mmol/L	Not reported	SD for 3 days post-implantation. Randomized to stay on SD or start KD with or without radiation on day 4. Radiation given to respective groups on days 3 and 5 post-implantation.
De Feyter et al. (18)	United States	Mouse	Post-implantation Day 11 90 mg/dL Day 15 90 mg/dL Day 18 115.2 mg/dL	Post-implantation Day 11 3.5 mmol/L Day 15 3.0 mmol/L Day 18 2.5 mmol/L	Not reported	KD started 7 days post-implantation.
Lussier et al. (20)	United States	Mouse	Not reported	Not reported	Not reported	SD for 3 days post-implantation. Randomized to stay on SD or start KD on day 4.
Rieger et al. (24)	Germany	Mouse	Not reported	KD alone: 1.7 mmol/L KD + chemotherapy: 1.4 mmol/L	Not reported	Randomized to KD or SD 7 days after implantation. Chemotherapy with diets started 12 days after implantation and given twice per week.
Mukherjee et al. (19)	United States	Mouse	KD-R: 90 mg/dL KD-R + DON: 100 mg/dL	KD-R: 1.3 mmol/L KD-R + DON: 1.2 mmol/L	Not reported	Restricted KD started 4 days after implantation. DON given at day 6, 8, 10, 12
Elsakka et al. (10)	Egypt	Human	Pre KD: 89 mg/dL Post-Surgery with KD: 72 mg/dL 3 monthS = 64 mg/dL 9 months = 75 mg/dL 15 months = 71 mg/dL 20 months = 65 mg/dL	Pre KD: Not reported Post-Surgery with KD: 3 mmol/L 3 months = 3 mmol/L 9 months = 0.7 mmol/L 15 months = 0.5 mmol/L 20 months = 0.7 mmol/L	Pre KD: 13.10 uIU/mL Post KD: 6.50 uIU/mL 3 months = 5.00 uIU/mL 9 months = 4.10 uIU/mL 15 months = 3.80 uIU/mL 20 months = 2.11 uIU/mL	Water fast for 3 days prior to surgery. Restricted KD for 21 days post water-fast prior to surgery. HBOT started 2 weeks post-surgery. No steroids administered but anti-seizure medications prescribed.
Woodhouse et al. (5)	United States	Human	Not reported	3 patients = ≥3 mmol/L 4 patients = 2.5 mmol/L 6 patients = 1.5 mmol/L 5 patients = 0.8 mmol/L 11 patients = 0.3 mmol/L	Not reported	MAD started before chemoradiation therapy. 6 weeks of MAD during chemoradiation therapy.
Zuccoli et al. (1)	Italy	Human	Before fasting/diet: 135 mg/dL At end of first fast: 90 mg/dL At end of second fast: 72 mg/dL At end of restricted KD: 63 mg/dL	Before fasting/diet: 0.0 mmol/L At end of first fast: 1.75 mmol/L At end of second fast: 2.5 mmol/L At end of restricted KD: 2.5 mmol/L	Not reported	Steroids and anti-seizure medications started prior to surgery and discontinued 30-days post-surgery. Patient did 2-day water fast post-surgery. Started KD and fasted 3 more days. Restricted KD for 14 days prior to chemoradiation therapy. Changed to calorie restriction only diet.
Artzi et al. (11)	Israel	Human	Not reported	Patient #1 and #4= >3 mmol/L Patient #2 and #5 = 3 mmol/L Patient #3 = 2 mmol/L	Not reported	Patients #2, #3, and #5 started KD at same time as chemotherapy. Patient #4 started KD a few months after chemotherapy. Patient #1 was only treated with KD for GBM. Patients #2, #3, and #4 received steroid treatment during KD with chemotherapy. Patients #2, #4 and #5 were intermittent with KD.
Champ et al. (8)	United States	Human	Time of Surgery: Patient #1 = 78 mg/dL Patient #2 = 146 mg/dL Patient #3 = 152 mg/dL Patient #4 = 135 mg/dL Other two patients not reported Max During Radiation: Patient #1 = 99 mg/dL Patient #2 = 90 mg/dL Patient #3 = 77 mg/dL Patient #4 = 103 mg/dL Other two patients not reported.	1 patient had 0.8 mmol/L 1 patient had 0.3 mmol/L All other patients not reported.	Not reported	Patient #1 started KD before chemoradiation Patient #2, #3 and #4 started KD during chemoradiation. Patient #5 and #6 started KD after chemoradiation. Patients #1, #2 and #4 received steroids during treatment.
Rieger et al. (24)	Germany	Human	Average Before KD: 99 mg/dL Average During KD: 92 mg/dL	7 patients = 1 mmol/L 5 patients = 0.8 mmol/L 2 patients = 0.4 mmol/L 1 patient had 0.3 mmol/L 1 patient was not reported	Not reported	8 patients started steroids before KD 11 patients started steroids during the KD
van der Louw et al. (23)	Netherlands	Human	Phase A (liquid diet): 5 patients 85 mg/dL 2 patients 80 mg/dL 2 patients 90 mg/dL Phase B (solid diet): 7 patients 90 mg/dL 1 patients 100 mg/dL 1 patient not reported	Phase A (liquid diet): 1 patient = 5 mmol/L 4 patients = 4 mmol/L 4 patients = 3 mmol/L Phase B (solid diet): 4 patients = 3 mmol/L 3 patients = 2 mmol/L 1 patient = 1 mmol/L 1 patient not reported	Not reported	KD for 2 weeks prior to treatment to reach ketones >3 mmol/L Chemoradiation administered for 6 weeks with KD KD only for 6 weeks followed by 1 cycle of chemoradiation End of study was regular diet with chemoradiation for 5 months

KD, ketogenic diet; SD, standard diet; BHB, β-hydroxybutyrate; GBM, glioblastoma multiforme; HBOT, hyperbaric oxygen therapy; DON, 6-diazo-5-oxo-L-norleucine.

When addressing glucose levels in patients with GBM, it should be discussed that some cancer therapies can increase glucose and glutamine levels in the brain. This increase can make it problematic to use the KD to deplete enough glucose while maintaining patient safety and diet tolerability. Patients with GBM may experience inflammation and swelling of the brain as part of radiation therapy (2–4). The increased swelling can lead to seizures and other neurological dysfunctions that can be detrimental to the patient (5). When this occurs, doctors prescribe steroids to control the swelling and inflammation (31). This is an unfortunate situation for the KD since steroid use increases glucose levels defeating the purpose of the KD (17).

Finally, many of the KDs implemented in these experiments used relatively low percentages of protein in addition to carbohydrates. Even though glucose is a primary driver of cancer metabolism, amino acids also have a strong presence in cancer cells to allow them to thrive. Many tumor cells rely on leucine, methionine, glutamine, and arginine to regulate growth and promote survival through cellular pathways such as mTOR and PI3K (32, 33). Since the average percentage of protein consumed in both the animal and human studies was around 14%, a decrease in amino acids may be a confounding factor for tumor response with the diets.

## 5. Conclusion

GBM continues to be one of the deadliest brain tumors affecting people of all ages across the world. The current treatment regimens are not proving to be effective in preventing disease progression or prolonging survival. As patients with GBM typically survive about 15 months after diagnosis, GBM is one of the most researched cancers to find better and safer treatments. With the current therapy consisting of surgery, radiation, and chemotherapy, this course of treatment exposes patients to further brain damage.

Since the 1920s, doctors and scientists have tested CR diets and KDs for treating many diseases and disorders. For example, the KD is well known for being studied in patients with epilepsy to help control seizures (7, 21). This phenomenon of ketones aiding in treatments is centuries old and continues to be explored in bounteous science disciplines. Cancer therapies and diet interventions display a defined connection to Otto Warburg and the Warburg Effect.

The testing of the KD has been well studied and documented in mouse models in various forms. Human studies using the KD continue to be small and challenging to research. All the studies included in this review showed mixed results on the KD enhancing tumor response to treatments and prolonging survival. Studies in animals have stronger results in reduced tumor growth and increased survival. Despite these promising findings, the methods used need to be consistent. Glucose and ketone levels as well as macronutrients need to be comparable. And the glioma cell lines used in mice need to be closer to the aggressive, vascular human GBM cell lines to represent real-life events.

It is worth mentioning that many of the mouse models only used diet interventions to treat the gliomas, which will not be the same for patients. Patients undergo intense radiation and chemotherapy after surgeons remove all or part of the tumor. These treatments cause further damage to the brain, and medications prescribed to reduce edema and inflammation can increase glucose levels defeating the sole purpose of a KD. These are just some considerations to factor into a study design when using a KD as a diet therapy in mice implanted with gliomas.

Based on the findings from this review, a KD is encouraging to be an effective form of adjuvant therapy with standard cancer treatment in adult patients with GBM. However, more reproducible results between pre-clinical and clinical studies are needed for a concrete decision on manageability and effectiveness. Future clinical studies should consider utilizing a nutritionist to design adequate caloric intake and macronutrient amounts for safe and optimal ketosis while providing motivational support. Researchers should also consider starting the KD well before surgery to ensure high ketone and low glucose levels are achieved before chemoradiation. Lastly, future pre-clinical research should focus on validating *in vitro* and *in vivo* methods that clearly define optimal concentrations of serum glucose and serum ketone levels to inhibit or disrupt cell signaling to create ideal adjuvant diet interventions with cancer therapies.

## Author contributions

The author confirms being the sole contributor of this work and has approved it for publication.

## Conflict of interest

The author declares that the research was conducted in the absence of any commercial or financial relationships that could be construed as a potential conflict of interest.

## Publisher’s note

All claims expressed in this article are solely those of the authors and do not necessarily represent those of their affiliated organizations, or those of the publisher, the editors and the reviewers. Any product that may be evaluated in this article, or claim that may be made by its manufacturer, is not guaranteed or endorsed by the publisher.
